# Supplementary material for: Biodegradable 3D Printed Scaffolds of Modified Poly (Trimethylene Carbonate) Composite Materials with Poly (L-Lactic Acid) and Hydroxyapatite for Bone Regeneration
Source: Nanomaterials (Basel). 2021 Nov 26;11(12):3215. doi: 10.3390/nano11123215 (PMC8706779; doi:10.3390/nano11123215)
Supplement: Supplementary file 1 [file nanomaterials-11-03215-s001.zip › nanomaterials-1438247-supplementary.pdf]

Supporting information

Biodegradable 3D Printed Scaffolds of Modified Poly (Trimethylene Carbonate) Composite Materials with Poly (L-Lactic Acid) and Hydroxyapatite for Bone Regeneration

Honglei Kang [1,2,†], Xudong Jiang [1,†], Zhiwei Liu [1], Fan Liu [1,*], Guoping Yan [1,*] and Feng Li [2]

[1] School of Materials Science and Engineering, Wuhan Institute of Technology, Wuhan 430205, China; kanghonglei@hust.edu.cn (H.K.); polymers2012@163.com (X.J.); polymers20121016@sohu.com (Z.L.)

[2] Department of Orthopaedics, Tongji Medical College, Huazhong University of Science and Technology, Wuhan 430022, China; lifengmd@hust.edu.cn

* Correspondence: Fan.liu@wit.edu.cn (F.L.); guopingyan@wit.edu.cn (G.Y.); Tel.: +86-27-65520-576

† These authors contributed equally to this work.

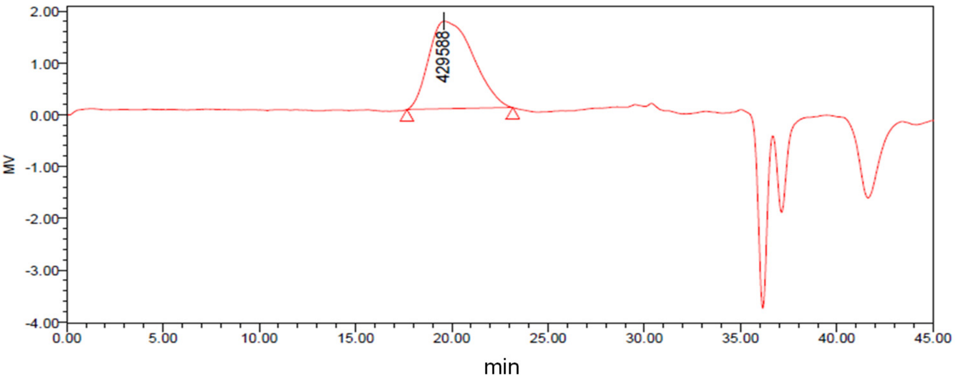

Mn	Mw	MP	Mz	Mz+1	Mw/Mn
251203	383046	429588	556214	740728	1.524846

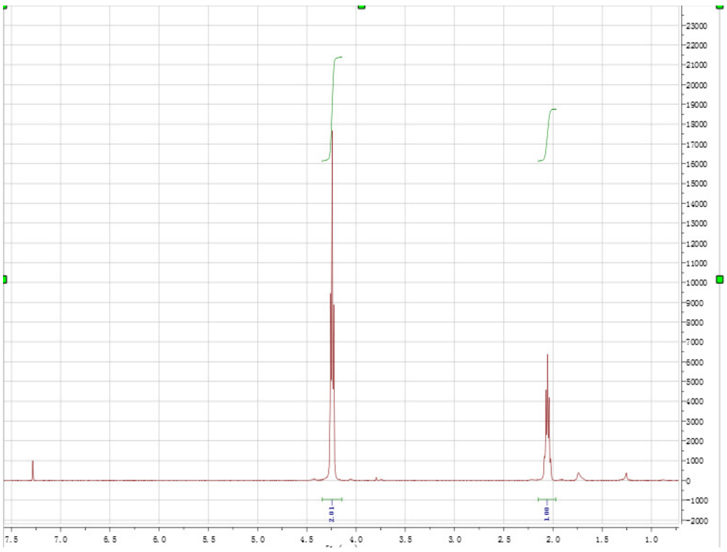

Fig. S1. GPC and ^{1}H NMR of PTMC and PLA
